# Knowledge, Attitude, and Practices of Schoolteachers Toward Epilepsy and Students With Epilepsy in the Aseer Region

**DOI:** 10.7759/cureus.49638

**Published:** 2023-11-29

**Authors:** Lama A Lahiq, Ibrahim Tawhari, Abdulaziz A Ojiman, Ahmed H Alshammari, Ahmed G Al ghanim, Faris S Alqahtani, Hatim A Asiri, Muhanned M AlObaid, Shahd K Abumilha, Abdulaziz A Alshahrani, Ahmed S AL Zomia

**Affiliations:** 1 College of Medicine, King Khalid University, Abha, SAU; 2 Internal Medicine, King Khalid University, Abha, SAU; 3 Pharm D Inventory Control Department, Rafha Maternity and Children Hospital, Rafha, SAU; 4 College of Medicine, King Khalid University, Riyadh, SAU; 5 Medical Affairs, Ministry of Interior, Jazzan, SAU

**Keywords:** misconceptions, first aid, inclusive education, attitudes, knowledge, teachers, epilepsy

## Abstract

Background: The knowledge that teachers have about epilepsy affects their attitudes and practices towards students with the disorder. This study aimed to explore teachers' knowledge, attitudes, and practices (KAP) toward epilepsy in the Aseer region.

Methods: This online cross-sectional survey targeted teachers aged 18 years and above, encompassing both males and females residing in the Aseer region with internet access. Teachers included in this study were recruited using snowball and convenience non-random sampling methods. Descriptive statistics and Pearson's chi-square test in Statistical Package for the Social Sciences (SPSS) version 27 were employed, with a significance level of 0.05 (IBM Corp., Armonk, NY).

Results: A total of 62 teachers were included in this study: 62.90% were aged 35-49, 93.55% were females, 90.32% were married, 80.65% worked in the governmental sector, 51.61% earned between 5,000 and 10,000 SAR, and 93.55% held a university education. Regarding knowledge, 36.6% of the teachers studied had very bad knowledge, 29% had poor knowledge, 35.5% had intermediate knowledge, and 4.8% had good knowledge. While 1.9% had a detrimental attitude about epilepsy, 58.1% had an incorrect attitude, 30.6% had an approximate attitude, and 1.6% had a correct attitude. Concerning teacher practice, 4.8% of the studied sample had detrimental practice, 56.5% had inadequate practice, and 4.8% had average practice.

Conclusions: The study highlights the need for targeted educational interventions to improve teachers' knowledge about epilepsy. The positive attitudes observed provide a foundation for fostering inclusivity in educational settings. Strategies that address misconceptions and improve first aid preparedness can contribute to a more supportive environment for students with epilepsy.

## Introduction

Seizures are one of the most common medical conditions in children, with epilepsy emerging as the most common chronic neurological condition among them [1]. Seizures are characterized by brief changes in consciousness that appear as particular behavioral and motor patterns brought on by an excessive discharge of electrical currents from a cluster of brain neurons [2]. Epilepsy is a brain disorder marked by a persistent tendency to experience epileptic seizures, which requires the occurrence of at least one such seizure [3]. In the general population, the prevalence of seizures is between 0.5% and 1%. Children with epilepsy have an incidence of 41-187/100,000. About 30,000 children and adolescents are diagnosed with epilepsy out of the approximately 150,000 who have their first unprovoked seizure each year [4]. In 2017, about 291 million children under 20 years of age had epilepsy and intellectual and sensory problems. The top ten countries represented 62% of children with these disabilities, and the remaining 95% live in low- and middle-income countries. [5]

A study in Saudi Arabia found that 6.54 out of 1000 individuals have epilepsy [6]. Another study looked at the prevalence of active epilepsy and its etiological variables in Saudi people living in Riyadh. Of the 13,873 individuals, 55 had active epilepsy between 2012 and 2016 (prevalence: 3.96/1,000 persons). The highest frequency was seen in infants, at 14.78/1,000 people. The most common types of partial seizures were complex ones, primarily caused by idiopathic and cryptogenic etiologies. The most common abnormalities to be diagnosed were localized, vascular, and structural [7].

Studies have indicated that a child's behavior, emotional and physical health, and academic performance are all adversely impacted by epilepsy [8-10]. Teachers are the first responders in the event that a seizure occurs in the school. Teachers' awareness of epilepsy and how to treat seizures can have a big impact on how well kids function, develop social skills, and maintain their general health [11].

The knowledge that teachers have about epilepsy affects their attitudes toward students who have the disorder. Even though the school might account for up to 40% of a child's developmental life, most instructors do not receive specific training on epilepsy during their schooling or training [12]. Teachers' attitudes regarding epilepsy refer to their proclivity to respond positively or negatively to various concerns concerning students with epilepsy. These attitudes shape their actions and reactions to challenges, incentives, and rewards [13]. While attitude is a complicated and ethereal concept, recent research has shown how teachers' attitudes can influence their actions, which can have negative effects on children who have epilepsy [14-16].

The study's hypothesis is that teachers in Saudi Arabia's Aseer region have a large disparity in their knowledge, attitudes, and practices (KAP) about epilepsy. We hypothesize that a number of social and demographic variables are important in determining how well-informed and cognizant teachers are about epilepsy. The lack of prior knowledge, attitude, and practice assessments of epilepsy among teachers in the Aseer region of Saudi Arabia is what drove this study. By recognizing these elements, we hope to identify certain domains in which educational interventions might be focused to improve teachers' understanding, attitudes, and practices toward epilepsy. The findings of this study are anticipated to provide valuable insights for the development of future community-based educational programs, specifically tailored for teachers, fostering a more informed and supportive environment for individuals with epilepsy.

## Materials and methods

Study setting

The study was conducted in the Aseer region of Saudi Arabia, an area with a population of 2,380,109 people, comprising 1,369,623 men and 1,010,485 women. The median age of residents in the Aseer Region is 32 years. Notable governorates in the region include Abha, Muhayil, An-Namas, Billasmar, Billahmar, Balqarn, Bareq, Bishah, Khamis Mushayt, Rijal Alma, Tathlith, Sarat Ubaidah, Ahad Rifaydah, Al-Majarah, and Al-Harajah.

Study design and participants

This online cross-sectional survey targeted teachers aged 18 years and above, encompassing both males and females residing in the Aseer region with internet access. The Aseer region hosts approximately 460,000 students across about 4,000 schools. The determined sample size, derived from a prior study indicating that 47% [17] of teachers possessed good knowledge about epilepsy, aimed to achieve a 95% confidence level, a margin of error of 0.05, and a power of 80%, resulting in a total of 50 teachers for the study. Teachers included in this study were recruited using snowball and convenience non-random sampling methods.

Data collection

The data collection framework for the study included a thorough evaluation of socioeconomic factors such as age, gender, place of residence, education, income, and marital status. The questionnaire, which was designed to collect data on epilepsy KAP, was based on previously published studies [18,19]. The questionnaire was uploaded to Google Forms and distributed via popular social media platforms such as Facebook Messenger and Telegram to reach a diverse audience. Strict measures were put in place to ensure data integrity. We implemented rigorous measures at every stage, including clear protocols for data collection, quality control checks, and validation procedures. Data storage security measures were enforced, and detailed documentation and training were provided to personnel. Each respondent was only allowed to submit one response. The work on KAP research by Essi and Njoya [20] was used to establish ratings for each section of the questionnaire. The categories are divided into three areas (knowledge, attitudes, and practice).

Knowledge

It consists of 18 questions with the following answers: yes, no, and I don’t know. Score (1) was given for the correct answer, while score (0) was given for the incorrect answer and I don’t know. The total score is 18, ranging from 0 to 18. Respondents were categorized as follows: very bad (50%); poor (50% and 65%); intermediate (65% and 85%); good (85%).

Attitudes

This section consists of seven questions. Answers were graded on a Likert scale ranging from strongly disagreeing (score 0) to strongly agreeing (score 5). The total score ranges from 7 to 35. Respondents were categorized based on total score into detrimental (50%); incorrect (50% and 65%); approximate (65% and 85%); and correct (85%).

Practices

This section consists of seven questions. The correct practice is given (1), while the incorrect practice is given 0. The total score is 7: detrimental (50%); inadequate (50% and 65%); average (65% and 85%); and adequate (85%).

Data analysis and statistical approach

The data underwent a thorough validation process, including checking for accuracy and consistency, before being cleaned and subsequently exported to an Excel file (Microsoft Excel, Microsoft® Corp., Redmond, WA). Within the Excel 2019 environment, figures were constructed to visually represent patterns within the dataset. For numerical data, a comprehensive statistical analysis was conducted, and the results were presented using key summary measures such as the mean and standard deviation. Meanwhile, for categorical data, a clear presentation was achieved through the use of counts and percentages, providing a concise and informative summary of the distribution of different categories. The collected data underwent analysis using Statistical Package for Social Science (SPSS, IBM Corp., Armonk, NY) version 27.

Ethical considerations

Before commencing the study, participants were comprehensively briefed on its objectives. Subsequently, each participant was required to provide written consent. Approval from the Research Ethics Committee at King Khalid University was sought. The research adhered rigorously to the principles outlined in the Helsinki Declaration throughout its duration.

## Results

Table 1 outlines the sociodemographic characteristics of the studied participants. A total of 62 teachers were included in this study. More than two-thirds of the participants fall in the 35-49 age group (62.90%), predominantly comprising females (93.55%). Married individuals constitute a significant portion (90.32%), while the governmental sector was the primary occupational category (80.65%). The income distribution shows a majority earning between 5,000 and 10,000 Saudi Riyal (SAR) (51.61%), and a substantial proportion holds a university education (93.55%). Residency was primarily in urban areas (80.65%) (Table 1).

**Table 1:** Sociodemographic characteristics of studied participants who were assed for their knowledge, attitude, and practices toward epilepsy and students with epilepsy in Asir Region (n = 62)

Studied variables	Level	Frequency	Percent
Age	18–24 years	1	1.61
	25–34 years	7	11.29
	35–49 years	39	62.90
	50–64 years	15	24.19
	65 years	1	1.61
Sex	Female	58	93.55
	Male	4	6.45
Marital status	Divorced	2	3.23
	Married	56	90.32
	Single	3	4.84
	Widow	1	1.61
Occupation	Governmental sector	50	80.65
	Private sector	4	6.45
	Retired	8	12.90
Income	Below 5000 SAR	4	6.45
	Between 15,000 and 20,000 SAR	23	37.10
	From 5,000 to 10,000	32	51.61
	More than 20,000 SAR	3	4.84
Education	University	58	93.55
	Post graduate degree	4	6.45
Residence	City	50	80.65
	Village	12	19.35

Table 2 presents key variables related to health insurance, smoking habits, and the presence of chronic diseases among the studied participants. Most participants lack health insurance (79.00%), with only 9.70% covered by government health insurance and 11.30% having private health insurance. Regarding smoking habits, a significant portion were non-smokers (94.60%), while a small percentage were ex-smokers (2.70%) or currently smokers (2.70%). In terms of chronic diseases, the majority did not have any (83.90%), while 16.10% reported having at least one chronic disease.

**Table 2:** Insights from health insurance, smoking habits, and chronic disease of studied participants who were assed for their knowledge, attitude, and practices toward epilepsy and students with epilepsy in Asir Region (n = 62)

Studied variables	Level	Frequency	Percent
Health insurance	Governmental health insurance	6	9.7
	Have no health insurance	49	79
	Private health insurance	7	11.3
Smoking	Ex-smoker	2	2.7
	Non-smoker	70	94.6
	Smoker	2	2.7
Chronic disease	No	52	83.9
	Yes	10	16.1

The table presents a comprehensive overview of the knowledge about epilepsy among the surveyed teachers. A substantial sector correctly identified epilepsy as a chronic disease of the brain (56.5%) and acknowledged that head trauma or brain infection can cause epilepsy (29%). Additionally, a significant proportion recognized that epilepsy is not a mental illness (61.3%), dispelling a common misconception. Furthermore, a majority correctly rejected supernatural explanations, with 58.1% dismissing the idea that epilepsy is caused by magic and an even higher percentage (88.7%) refuting the idea that it can be transmitted through physical contact or bodily fluids. Importantly, a positive perspective emerged regarding the treatability and control of epilepsy, with 62.9% acknowledging that it can be managed. Furthermore, the majority (58.1%) correctly recognized the dependence of epilepsy treatment on modern medicine rather than traditional methods (41.9%) (Table 3).

**Table 3:** Teacher knowledge regarding epilepsy

Knowledge question		n	%
Do you think epilepsy is a chronic disease of the brain?	Don’t know	19	30.6
	No	8	12.9
	Yes	35	56.5
Can head trauma or brain infection cause epilepsy?	Don’t know	30	48.4
	No	14	22.6
	Yes	18	29
Do you think epilepsy is a mental illness?	Don’t know	14	22.6
	No	10	16.1
	Yes	38	61.3
Do you think epilepsy is caused by magic?	Don’t know	20	32.3
	No	6	9.7
	Yes	36	58.1
Do you think epilepsy is caused by demon acquisition?	Don’t know	23	37.1
	No	7	11.3
	Yes	32	51.6
Do you think students with epilepsy usually have associated mental retardation?	Don’t know	20	32.3
	No	2	3.2
	Yes	40	64.5
Do you think students with epilepsy have below-average school performance?	Don’t know	21	33.9
	No	10	16.1
	Yes	31	50
Do you think genetics are the main cause of epilepsy?	Don’t know	24	38.7
	No	25	40.3
	Yes	13	21
Do you think students with epilepsy have under-average intelligence?	Don’t know	26	41.9
	No	14	22.6
	Yes	22	35.5
Do you think students with epilepsy have a high risk of going crazy?	Don’t know	27	43.5
	No	7	11.3
	Yes	28	45.2
Do you think epilepsy is an infectious disease?	Don’t know	20	32.3
	No	6	9.7
	Yes	36	58.1
Do you think epilepsy is transmitted through physical contact with someone with epilepsy, their saliva or urine?	Don’t know	4	6.5
	No	3	4.8
	Yes	55	88.7
Do you think that epilepsy is transmitted by contact with the place where the person fell during the seizure?	Don’t know	7	11.3
	No	4	6.5
	Yes	51	82.3
Do you think epilepsy can be treated or controlled?	Don’t know	17	27.4
	No	6	9.7
	Yes	39	62.9
Does the treatment of epilepsy depend on modern medicine?	Don’t know	22	35.5
	No	4	6.5
	Yes	36	58.1
Does the treatment of epilepsy depend on traditional medicine?	Don’t know	28	45.2
	No	8	12.9
	Yes	26	41.9

**Figure 1:**
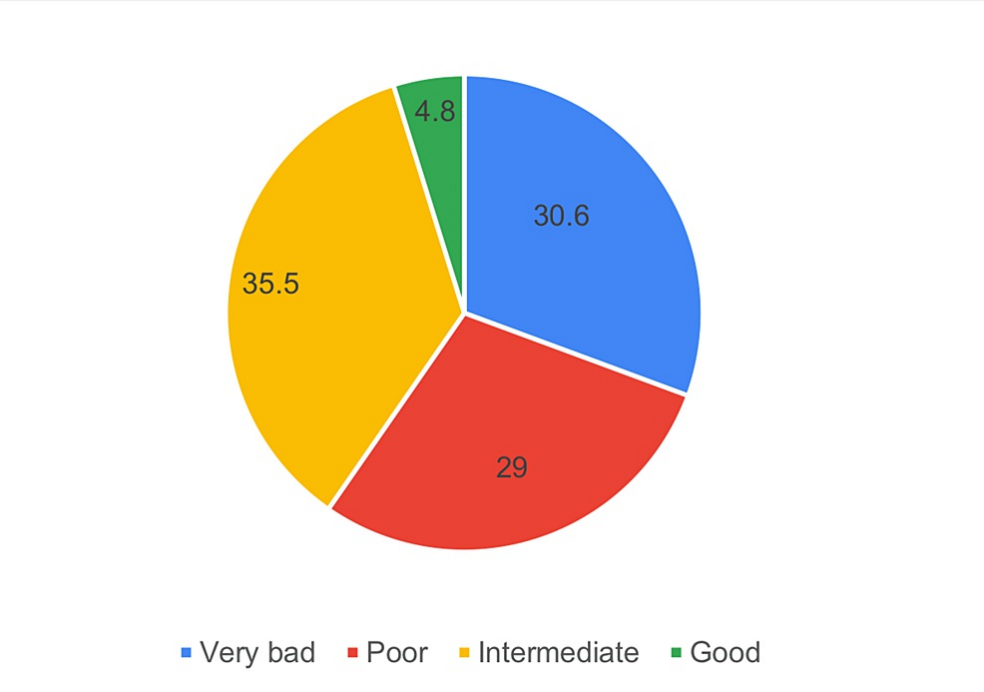
Distribution of the study participants according to the level of knowledge about epilepsy

Figure 1 shows that 36.6% of the studied teachers had very bad knowledge, 29% had poor knowledge, 35.5% had intermediate knowledge, and 4.8% had good knowledge. In general, 40.3% scored above 65%.

Participants predominantly endorse inclusivity, with 27.5% believing that exercise should not be strictly forbidden for children with epilepsy. A substantial sector (53.3%) considers a student with epilepsy controlled by medications to be on the same level as students with other chronic diseases, fostering an inclusive mindset. Additionally, a large proportion (43.5%) rejected the idea that having a student with epilepsy in class can disrupt the learning process, indicating a positive attitude toward integration. Furthermore, a significant portion (27.4%) disagreed with the idea that having a student with epilepsy can have adverse psychological effects on other students. In terms of social interactions, 69.3% expressed willingness to allow their child to play with or sit with a child with epilepsy, demonstrating acceptance. Nearly two-fifths, or 38.7%, also rejected the idea of discriminating against individuals with epilepsy in marriage (Table 4).

**Table 4:** Attitude of teachers about epilepsy

Attitude questions	Level	n	%
Do you think that exercise should be strictly forbidden for children with epilepsy?	Strongly disagree	4	6.5
	Disagree	13	21
	Neutral	19	30.6
	Agree	13	21
	Strongly agree	13	21
Do you think a student with epilepsy controlled by medications is like any other student with another chronic disease?	Strongly disagree	2	3.2
	Disagree	10	16.1
	Neutral	17	27.4
	Agree	12	19.4
	Strongly agree	21	33.9
Do you think having a student with epilepsy in your class can disrupt the learning process in the classroom?	Strongly disagree	11	17.7
	Disagree	16	25.8
	Neutral	14	22.6
	Agree	14	22.6
	Strongly agree	7	11.3
Do you think having a student with epilepsy in class can have bad psychological effects on other students?	Strongly disagree	9	14.5
	Disagree	8	12.9
	Neutral	20	32.3
	Agree	15	24.2
	Strongly agree	10	16.1
Do you think students with epilepsy should be placed in a class dedicated to them?	Strongly disagree	17	27.4
	Disagree	11	17.7
	Neutral	15	24.2
	Agree	11	17.7
	Strongly agree	8	12.9
Will you allow your child to play or sit in the same row with a child with epilepsy?	Strongly disagree	2	3.2
	Disagree	3	4.8
	Neutral	14	22.6
	Agree	19	30.6
	Strongly agree	24	38.7
Will you allow your child to marry someone who suffers from epilepsy?	Strongly disagree	4	6.5
	Disagree	16	25.8
	Neutral	18	29
	Agree	13	21
	Strongly agree	11	17.7

**Figure 2:**
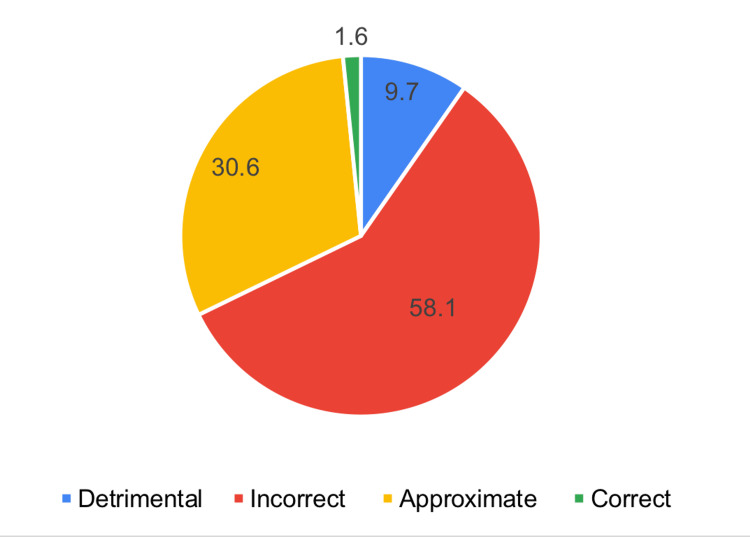
Distribution of the study participants based on their attitude towards epilepsy

Figure 2 shows that 1.9% had a detrimental attitude about epilepsy, 58.1% had an incorrect attitude, 30.6% had an approximate attitude, and 1.6% had a correct attitude. In general, 32.2% had scored above 65.

In response to the question of providing first aid to a student experiencing an epileptic seizure in class, the majority (75.80%) expressed a positive practice, indicating their willingness and capability to offer assistance. On the other hand, when it comes to offering any type of prescribed treatment, such as tassels, during a seizure in class, opinions are more evenly divided. More than half (54.80%) indicated a reluctance to administer prescribed treatment, possibly due to concerns about the appropriateness or efficacy of such interventions in a classroom setting. Two-thirds of teachers (67.74%) correctly acknowledged that when a person experiences an epileptic seizure, it is not advisable to put something in their mouth to keep the airway open. Additionally, a significant proportion (79.03%) recognized the importance of keeping the person by their side until the seizure passed. Moreover, the majority of teachers (70.97%) correctly believed that it is preferable to clean the airway if a child with epilepsy is not breathing well during a seizure. In terms of post-seizure actions, a substantial number of teachers (67.74%) correctly indicated that the child should be taken to the hospital at the end of the crisis, demonstrating a responsible and informed approach (Table 5).

**Table 5:** Teacher practices with children having epilepsy

Practice questions		n	%
When a person has an epileptic seizure, it is best to put something in their mouth to keep their airway open	No	20	32.26
	Yes	42	67.74
When a person has an epileptic seizure, it is best to keep them on their side until the crisis passes	No	13	20.97
	Yes	49	79.03
When a child has an epileptic seizure, it is preferable to clean the airway if the child is not breathing well	No	18	29.03
	Yes	44	70.97
At the end of the crisis, the child should be sent home	No	25	40.32
	Yes	37	59.68
At the end of the crisis, the child should be taken to the hospital	No	20	32.26
	Yes	42	67.74
Can you can provide first aid to a student who has an epileptic seizure in class?	No	15	24.19
	Yes	47	75.81
Can you offer any type of prescribed treatment, such as tassels, to a student who has an epileptic seizure in class?	No	34	54.84
	Yes	28	45.16

**Figure 3:**
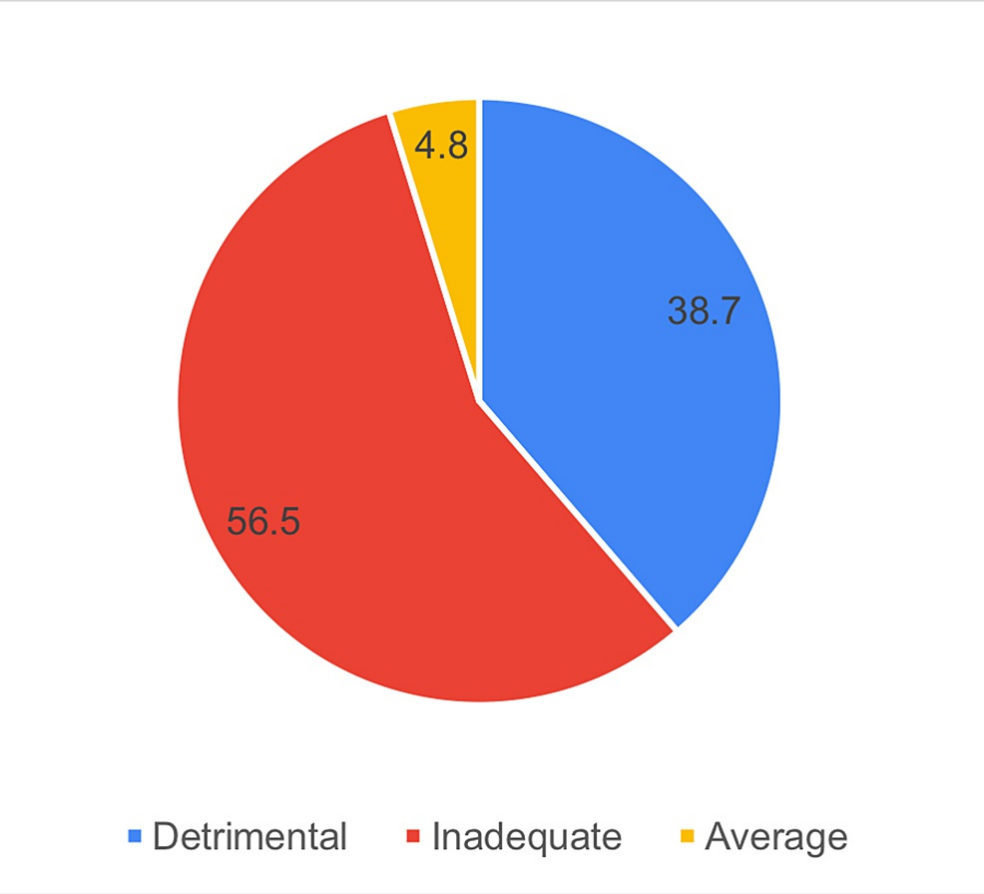
Distribution of the study participants according to their practice about dealing with a child with epilepsy

Figure 3 shows that 4.8% of the studied sample had detrimental practice, 56.5% had inadequate practice, and 4.8% had average practice.

## Discussion

Main study findings

The study aims to assess the knowledge, attitudes, and practices of teachers in the Aseer region, Saudi Arabia, regarding epilepsy. Regarding knowledge, 36.6% of the teachers studied had very bad knowledge, 29% had poor knowledge, 35.5% had intermediate knowledge, and 4.8% had good knowledge. Of the studied teachers, 56.5% identified epilepsy as a chronic brain disease, 29% recognized head trauma or brain infection as potential causes, 61.3% dispelled the misconception that epilepsy is a mental illness, 58.1% rejected supernatural explanations, and 88.7% dismissed the idea that epilepsy can be transmitted through contact or bodily fluids. Positive perspectives on its treatability emerged, with 62.9% acknowledging its manageability and 58.1% recognizing the reliance on modern medicine. Concerning attitude, 1.9% had a detrimental attitude about epilepsy, 58.1% had an incorrect attitude, 30.6% had an approximate attitude, and 1.6% had a correct attitude. Specifically, 27.5% believed exercise should not be forbidden for children with epilepsy, 53.3% considered students with epilepsy controlled by medications on par with those having other chronic diseases, and 43.5% rejected the idea that having a student with epilepsy disrupts learning. 27.4% disagreed with adverse psychological effects, 69.3% expressed willingness for their child to interact with a child with epilepsy, and 38.7% rejected discriminating in marriage. Concerning teacher practice, 4.8% of the studied sample had detrimental practice, 56.5% had inadequate practice, and 4.8% had average practice. In response to providing first aid during a seizure, 75.80% express a positive practice, while 54.80% are divided on offering prescribed treatments, possibly due to concerns about their appropriateness in a classroom setting. Overall, these findings underscore the need for targeted education to enhance knowledge and foster inclusive attitudes among teachers regarding epilepsy.

Interpretation of the main study findings

In the context of this study, the findings reveal that 40.3% of participants achieved scores surpassing 65% on the knowledge scale. Additionally, 32.2% of respondents demonstrated a positive inclination, scoring above 65 on the attitude scale. On the practical front, a smaller percentage, 4.8%, scored above 65% on the practice scale, highlighting a more modest proportion engaging in practices reflective of a high level of proficiency. Similar findings were reported by a study conducted in Taif City, Saudi Arabia, involving 420 schoolteachers. The study found that all participants were familiar with epilepsy, and 88.1% believed that an epileptic fit equated to seizures. Interestingly, 78.6% expressed dissatisfaction with their level of knowledge, and the practice score was 6.9 out of 15. Yet 50.2% admitted to feeling fearful about having an epileptic child in their class [21]. A similar study was conducted by Babikar and Abbas [17]. They assessed the knowledge, attitude, and practice of 200 school teachers regarding epilepsy. The majority of respondents had never been informed about the disorder, giving evasive answers. Few considered epilepsy contagious, and none objected to having epileptic children in their classes. Of the studied teachers, 47% of primary school teachers had knowledge of initial procedures for helping a seizure child, compared to 64% in secondary schools. Another study assessed the knowledge, attitude, and practice of secondary school teachers in Assiut, Egypt, regarding epilepsy. Findings revealed that all teachers were aware of epilepsy, and 54% treated students with epilepsy as normal, and 12.7% accepted giving the student, who was having a seizure in the class, any form of prescribed treatment [19].

This study observed a positive attitude among teachers toward several crucial aspects of epilepsy, such as dispelling associations with magic and demons. Furthermore, the research identified positive knowledge and attitudes regarding significant aspects, including the perception of epilepsy as a potentially curable mental condition. However, a survey involving 845 teachers from 20 schools in three sub-cities of Addis Ababa revealed a contrasting perspective, where a notable proportion of teachers considered epilepsy as a psychiatric illness linked to insanity, aligning with prevailing cultural beliefs. The recommendations for remedies, such as holy water treatment and church healing sessions, were consistent with this perception [18]. This discrepancy in attitudes underscores the need for targeted educational interventions to foster a more accurate and informed understanding of epilepsy among teachers, aligning their perspectives with medically grounded information.

Despite the higher prevalence of good knowledge, we found poor practice among the Saudi teachers included in this study. Similarly, a study was conducted in the northern region of Aljouf, Saudi Arabia. Teachers would do the following to a seizing child: 52% would pull the child's tongue out, 21.6% would put a spoon in the child's mouth, 14.1% would remove any tight clothing, and 79.7% would take the child to the hospital. The study concluded that the majority of Aljouf schoolteachers had adequate knowledge about students with epilepsy, but they needed more information about how to practice with students with epilepsy [14].

Implication for future research

These findings have far-reaching implications for both education and healthcare. To begin, identifying knowledge gaps in teachers highlights the need for targeted educational programs to improve their understanding of epilepsy, dispel myths, and foster accurate information. This can help to create a more supportive and inclusive environment for students with epilepsy, as well as reduce stigma and discrimination. Furthermore, the positive attitudes observed in many aspects suggest that teachers are willing to embrace inclusivity, which can be fostered further through awareness campaigns and training. Recognizing that a significant proportion of teachers are willing to provide first aid during a seizure is critical in healthcare. This points to the possibility of implementing first aid training programs in schools, providing educators with practical skills to support students with epilepsy.

Strengths and limitations

The strengths of the research include its comprehensive exploration of teacher knowledge and attitudes toward epilepsy, providing valuable information on potential areas for improvement in education and healthcare practices. Additionally, the study covers various aspects, including knowledge about epilepsy, attitudes towards students with epilepsy, and willingness to provide first aid, providing a holistic view of teachers' perceptions. However, there are some limitations to consider. The research relies on self-reported data, which may be subject to social desirability bias, where participants provide responses they believe are socially acceptable. The small sample size and use of the non-random sampling method limit the generalizability of the findings to a broader population.

## Conclusions

A large percentage of Saudi teachers were knowledgeable about epilepsy. They correctly identify epilepsy as a chronic brain disease and its potential causes, while debunking myths about its nature. Many teachers support inclusivity, reject stigma, and are eager to help students with epilepsy. Despite a significant proportion of good knowledge, there are still significant gaps, indicating the need for targeted educational interventions. Teachers generally have positive attitudes, challenge stereotypes, and foster an accepting environment. In terms of practical responses, the majority were inadequate. They were, however, willing to provide first aid during a seizure, indicating a supportive stance, whereas opinions on prescribing treatments are more evenly divided. These findings emphasize the importance of targeted interventions and educational initiatives aimed at increasing teachers' knowledge, correcting misconceptions, and improving epilepsy practices. Addressing these issues is critical to creating a more informed and supportive environment for students with epilepsy in educational settings.
